# Automatically annotating documents with normalized gene lists

**DOI:** 10.1186/1471-2105-6-S1-S13

**Published:** 2005-05-24

**Authors:** Jeremiah Crim, Ryan McDonald, Fernando Pereira

**Affiliations:** 1 Department of Computer and Information Science, University of Pennsylvania, Levine Hall, 3330 Walnut Street, Philadelphia, Pennsylvania, USA, 19104

## Abstract

**Background:**

Document gene normalization is the problem of creating a list of unique identifiers for genes that are mentioned within a document. Automating this process has many potential applications in both information extraction and database curation systems. Here we present two separate solutions to this problem. The first is primarily based on standard pattern matching and information extraction techniques. The second and more novel solution uses a statistical classifier to recognize valid gene matches from a list of known gene synonyms.

**Results:**

We compare the results of the two systems, analyze their merits and argue that the classification based system is preferable for many reasons including performance, simplicity and robustness. Our best systems attain a balanced precision and recall in the range of 74%–92%, depending on the organism.

## Background

Recently, researchers have begun to apply machine-learning-based information extraction techniques to biomedical text, most notably to identify gene and chemical compound mentions [1-3]. A related problem is gene normalization, which involves annotating a document (or abstract) with the list of identifiers for genes mentioned in the document. Gene normalization requires as input a *synonym list*. Each entry in the list represents a specific gene and contains both a unique identifier for that gene, called here a normal form, and a set of different ways in which the gene has been or may be mentioned. Ideally this list will be complete. However, in practice, gene mentions vary widely and evolve over time. Table 1 contains a few entries of the synonym list for the fly organism. To facilitate gene normalization research, the organizers of BioCreative [4] have made available a number of abstract/normalized-list pairs for three different organisms: fly, mouse and yeast. The exact breakdown is 5000 for training, 100–250 for development (depending on the organism) and 250 for evaluation. They also provided extensive synonym lists based on resources from the associated model organism databases.

The problem of gene normalization is relatively new and unexplored. Morgan et al. [5] provide a good introduction to the problem space and present a two staged system that identifies mentions and then labels each mention with a normal form. The focus of their work is on the fly organism.

Gene normalization is both easier and harder than identifying gene mentions. It is easier because it does not require textual boundaries of each mention to be identified, but only that some mention be detected and the document annotated accordingly. On the other hand, gene normalization is harder than identifying mentions in that it requires the actual gene to be detected and associated with the organism-specific unique gene identifier. The three organisms under consideration, yeast, fly, mouse, have from thousands to tens of thousands of genes. Even if it were possible to identify every gene mention with 100% accuracy, it would still be difficult to disambiguate each mention given the number of possibilities and the high degree of overlap among synonym lists for different but related genes (see Hirschman et al. [6] for an analysis of number of terms and degree of ambiguity for this task).

Gene normalization can thus be described by its two fundamental difficulties. The first is finding those mentions that do not occur as a known synonym in a curated list. This problem is highly correlated to the recall of the system: a more extensive synonym list, or a better way of inferring that some mention pertains to a gene in the list, increase the chance that the system will recognize a gene that is mentioned in the text. The second problem is to determine which unique gene each identified mention refers to. This is correlated with the precision of the system in that some identified mentions may not actually be genes, or might be genes for other organisms or might be ambiguous amongst many genes. Identifying that these mentions do not match a unique identifier will reduce the amount of incorrect genes returned by the system.

The two systems we present here primarily focus on the second problem: identifying the unique gene that has been mentioned. This includes identifying whether some mention is erroneous. In general the systems treat the given synonym lists as complete, with some tolerance for punctuation and orthographic differences. Even with this assumption we were able to achieve good performance for all organisms: 74%-92% balanced precision and recall. The first system presented relies on a series of pattern matching techniques to find and filter synonym string matches. The second system extracts all possible synonym matches and uses a binary classifier to determine which are valid.

### Initial directions

With the availability of highly accurate gene taggers [2], one simple approach would be to extract all the gene mentions from text and to match these mentions to the synonym list of each organism. However, there are many difficulties with such an approach. The primary problem is that mentions may be ambiguous. For instance, the gene mention *alcohol dehydrogenease *is a valid synonym for 111 different fly genes. Simply matching *alcohol dehydrogenease *to all 111 genes would lead to a steep decline in precision (since the mention is most likely referring to only one specific gene). Second, the system would be dependent on the accuracy of the gene tagger. Our experiments showed that for mouse, the gene tagger used [2] performed reasonably well on the development data. However, for fly and yeast, the tagger's performance was less than useful. This is most likely a result of the fact that the tagger training data did not contain enough examples for those organisms. These experiments were conducted by visual inspection on the tagged output of the gene tagger. No exact numbers of tagging performance can be provided on these data sets since the data is not internally annotated with gene mentions.

The above approach was taken by Morgan et al. [5] for the fly organism and their system achieved very promising results: 88% precision and 61% recall. The system is two staged. The first uses an HMM gene tagger to find mentions of genes in text. The second step looks for matches of each mention to a known synonym while carefully filtering those matches on highly ambiguous or unreliable synonyms. The primary reason that this approach worked for Morgan et al. is that they were able to create a training set specific to fly for identifying gene mentions. This set was created by reverse engineering of the synonym list with a set of gene normalized training documents to find gene mentions in each document. The training data is noisy, but experiments run by Morgan et al. show the trained tagger to perform at a reasonable level. 

Another seemingly straightforward approach is to treat the problem as multi-class document classification. Here, each normalized gene form is a possible class and the goal is to label each document with a set of classes (genes). We encountered two major problems to this approach. Multi-class document classification is typically done for tens and in rare instances hundreds of classes. However, as stated earlier, each organism has thousands of genes and in some cases tens of thousands. This poses substantial computational issues. Another major obstacle is that not all classes are observed in the training data. Only 22%, 13% and 47% of all fly, mouse and yeast genes were ever seen in the training data. This would make it impossible to gather the enough statistics to make accurate predictions.

## Implementation

In this section we present the implementation details of our two approaches to gene normalization.

### Pattern matching

Given a synonym list that is both unambiguous and exhaustive, creating a normalized gene list would be trivial. We could simply match every occurrence of a synonym in the text, and based on those matches label the document with the corresponding normalized mentions. Unfortunately, the synonym list for this task has neither property. As previously mentioned, many synonyms are ambiguous, either occurring with multiple genes or in contexts where no gene mentions are present. For instance, *blink*, *with *and *weak *are all listed in the fly synonym list. Matches to these common English words will most likely not constitute actual gene mentions. But even with these ambiguities, which increase greatly the number of genes that a simple pattern matching system would propose, using the synonym list does not retrieve all mentions. This does not mean that a pattern matching approach is useless, our first system relies heavily on standard techniques. However, we do not assume that every synonym in the list reliably labels documents with their normalized gene mentions. Instead, we prune each organism's synonym list so that it only contains synonyms that we believe will be informative, based on labeled training documents. A synonym, *s*, for a gene, *g*, is considered informative if and only if for the training set *D*:



The left-hand-side fraction is the conditional probability of *g *labeling a document, given that there was a match of *s *in the document. The threshold *δ *was tuned to 0.4 on the development data set for all three organisms. The result was that the number of total synonyms was reduced from 99501 to 4029 for fly, 130548 to 2898 for mouse and 14756 to 2307 for yeast. This reduction is substantial, since, as Table 2 indicates, it resulted in an absolute increase in f-measure of 50% for fly and 27% for mouse. *δ *is directly proportional to precision and recall. Higher values of *δ *result in higher precision at the expense of recall and lower values of *δ *result in higher recall at the expense of precision.

While using a pruned synonym list performs significantly better than simple pattern matching with the original list, the system still predicts far too many genes for each document. To further restrict the genes considered, a second stage of the pattern matching system produces, for each document, a set of candidate genes. Now, only genes that are present in the candidate list for a document and are also associated with an informative synonym in that document will be added to the document's final list.

For a fly-related document, the system extracts the 1000 closest documents in the training data using Euclidean distance over word indicator feature vectors:



*I*(*w*, *d*) = 1 if word *w *is in document *d *and 0 otherwise

Only words that did not occur in a stop-list of common English words were included in this calculation. The gene lists for the neighbouring documents are merged to create the final candidate list.

For mouse-related documents, the system first tags the document using a gene tagger [2]. Each gene mention is then compared to every synonym in the mouse synonym list. If the gene mention and a synonym have a Jaro-Winkler similarity [7] greater than 0.85, then the gene that synonym is associated with is added to the candidate list for that document. The Jaro-Winkler metric returns a similarity score in the 0 to 1 range based on many factors, including the number of characters in common (shared by both strings and in the same order) and the longest common substring, both of which stress the importance of characters occurring in the same order.

This two-stage pattern matching system compensates for the fact that the given synonym list contains large amounts of ambiguity, but does nothing to reduce the number of gene mentions that a naive pattern matching approach misses due to the incompleteness of the synonym lists. We observe that many of these omissions occur because of differences in punctuation or morphology. Thus, the pattern matching system includes a third, and final stage. In it, all punctuation is removed and each token is stemmed with the Porter stemmer [8] in both the documents and the synonym lists. Stemming removes missed matches due to minor morphological differences like, *human beta-globin peptides *versus *human beta-globin peptide*. As before, each informative synonym is compared to each document. If the synonym matches and the corresponding gene is in the candidate list for that document, then the gene is added to that document's final gene list.

Finally we will note that the yeast system required neither a candidate list nor stemming to gain maximum performance. The reason for this is that most researchers refer to yeast genes using a standard nomenclature, where each gene is usually mentioned in one of a few known and unambiguous forms. Table 2 shows the performance for the different stages of the system for the fly and mouse organisms. Table 3 summarizes the final results of the system on the evaluation data.

### Shortcomings of pattern matching

It is rather surprising how well pattern matching can do if used with some care. However, there is something unsettling about this approach. First, several parameters need to be adjusted on the development data. These include *δ *as well as the various parameters required to create the neighbour lists (for instance, number of closest documents and the Jaro-Winkler distance). For all hand-tuned parameters there is the danger of overfitting to the development data. By examining Tables 2 and 3, it appears that this may not be a problem for this set of data. In particular, the F1 score for the mouse organism is actually better on evaluation then on development, and for both fly and mouse the recall on the evaluation set is higher than the recall on the development set. Nevertheless, these hand-tuned parameters are difficult to set and there is no supporting theoretical analysis for their effect.

The complexity of the system is also a problem. Data is transformed in many stages: stemming, creating the informative synonym list, creating the neighbor list and finally matching the synonyms to the text. As with all pipelined systems, this may lead to cascading errors in which an error early in the pipeline will cause errors to be made at later stages.

A lack of uniformity between each organisms system is also an undesirable trait. Particularly, neighbor lists are generated differently for each organism or not at all in the case of yeast. One could easily argue that the method will not generalize well to other organisms. What we really desire is one uniform approach, for all organisms, in which every parameter is automatically set during the training phase.

### Match classification

Consider a system in which every time a synonym had a text based match with a document the system also labeled that document with that synonyms normal form. The inspiration for our second model comes from the observation that this kind of liberal pattern matching can achieve a very high recall (91%, 79% and 90% for fly, mouse and yeast on the development data). The problem, as addressed in the last section, is that this also results in extremely poor precision. However, just as it is possible to use the training data to determine which synonyms are useful, it is also possible to use the training data to determine which matches are correct.

We present here a model that, given a set of synonym matches, distinguishes correct from incorrect ones. This is essentially a binary classifier in which good matches are positively labeled and bad matches negatively labeled. To create training data for the classifier, we matched every synonym to each training document using a loose matching criterion (punctuation and numbers were ignored). We then extracted, for each match, the text that matched (adding back the removed characters), some context of the match, the normal form causing the match, as well as the number of other genes which matched that specific piece of text. For the training data, if the normal form for a match was in the normalized gene list for that document, then the match was labeled positive. Below are some examples of such matches:

of drosophila *Kinesin heavy chain *attached to, FBgn0001308, 1, Y

was analyzed *in *trajectories with, FBgn0001250, 5, N

homeotic gene *Ultrabithorax *(ubx, FBgn0013100, 7, N

The italicized text is the text causing the match. We extract two words before and after the match. In the first example, the normalized form causing the match is FBgn0001308, it was the only gene matching that piece of text and it constituted an actual match. Note that the third match, *Utrabithorax*, is negative because it is actually a match for the gene FBgn0003944, which shares the synonym *Utrabithorax *with FBgn0013100.

This provided a large set of positive and negative matches required to train a classifier. We used the MALLET [9] implementation of maximum entropy models [10] for our classifiers. Maximum entropy classifiers model the conditional probability of a class given an input vector with the log-linear form:



where *y *is a class (in our case *yes *or *no*), **x **is an input vector and *Z*(**x**) is a normalizing term. (It is easy to see that in the binary case, maximum entropy classification is equivalent to logistic regression.) For our model, **x **is a binary vector containing predicates on the matched text, its context, the normal form causing the match and the number of other genes matching the text. Each feature function *f*_*i*_(*y*, **x**) maps an input vector and class to a binary variable, for instance:



The above feature has a value of 1 for all matches in which the word directly before the matched text is *drosophilia *and the classification for the match is *yes*. The parameters of the model are the feature weights *λ*_*i*_. Ideally one would like the weights of features that tend to be on for correct classifications to be strongly positive, the weights of features that tend on for incorrect classifications to be strongly negative, and the weights of uninformative features to be zero. To accomplish this the parameters are set to maximize the log-likelihood of the training data :



A Gaussian prior over weights, with variance tuned to 1.0 on the development data, reduces the danger of overfitting the model to the training data [11]. The log-likelihood function is concave allowing optimal parameter values to be found by numerical optimization methods. Our system uses a limited-memory quasi-Newton method, which has been shown to be one of the most efficient algorithms for maximizing the log-likelihood of maximum entropy models [12,13].

To classify a new abstract, the system first extracts all the synonym matches that occur within it. Then for each match **x**, the classifier finds the classification *y *∈ {*yes*, *no*} with the highest probability *P*(*y*|**x**). For each positive match, the normal form causing the match is added to the documents normalized gene list. Results of the trained models on evaluation data are shown in Table 4. The evaluation results differ from the official BioCreative 2004 results. This is due to a match extraction error that was discovered and resolved after the official results were submitted.

A quick glance at the results show that maximum entropy classification does as well or better than pattern matching for all organisms without the need for organism specific optimizations. The only tunable parameter in this models is the Gaussian prior. However, most experiments suggest that performance does not change dramatically with different values for the prior. The prior exists primarily to keep weights as close to zero as possible and in all our experiments setting the prior to 1.0 was satisfactory to prevent overfitting.

## Results

Tables 3 and 4 outline the results for both the pattern matching and the maximum entropy classification systems. The results are comparable for mouse and yeast, however, the classification system outperforms the pattern matching system for fly-related documents. The best system for all organisms performs reasonably well with performance ranging from 74% to 92%.

The primary limitation of the pattern matching and maximum entropy classification models are their reliance on the gene synonym list to find potential matches. For the three organisms under consideration here, it is possible to attain a maximum recall of 91%, 79% and 90% for fly, mouse and yeast using the their synonym lists to match the text. In theory, if the synonym lists were complete we could see an increase of up to 20% in recall. This problem may be exacerbated for other organisms that do not have synonym lists that have been as well curated as the ones for fly, mouse and yeast.

A simple mechanism to increase the recall for synonym matching is to relax the matching criteria further using some sort of edit distance or string similarity metric [7]. Recently, Tsuruoka and Tsujii [14] have experimented with soft matches for protein name recognition. The difficulty with such an approach is that the matching algorithm would be potentially less efficient, with a naive *O*(*n*^2^) implementation being totally impractical for this task. However, approximate string matching techniques as developed in computational biology might be useful here.

Another approach could be to generate these synonym lists automatically. Work on this problem has been done by Yu and Agichtein [15]. However, the results of their system are below 50% F1 measure and may not yet be usable in practical applications such as gene normalization.

## Conclusion

Comparing Tables 3 and 4, we see that maximum entropy classification does just as well or better than the pattern matching system. A primary advantage of maximum entropy classification over pattern matching is that the system is uniform across organisms, hence the method is more likely to perform well when extended to different organisms.

There are many ways in which the maximum entropy model can also be improved. The most obvious of which is to include more expert knowledge into the model. Maximum entropy models are widely used since they easily allow for the integration of such expert knowledge through the definition of new features. For extracting gene mentions from text, these features generally take the form of lexical resources and indicative regular expressions [2]. For gene normalization, it may be possible to have experts additionally curate the synonym list to indicate which synonyms should be trusted and which should not. This could greatly improve performance, particularly for synonym matches not seen in training. If the system matches have a feature indicating that a synonym is trustworthy it could provide evidence to classify the match as valid. Currently, the model's features are based primarily on textual matching and contain no domain specific information. It may also be possible to improve performance by introducing more context or some syntactic features from the extracted matches. However, preliminary experiments on the development data suggested that additional context had a negligible effect on accuracy and only served to increase the time it took to train the model.

Another potential improvement would be to relax the criteria when extracting matches. Under perfect conditions we should be able to extract all good matches and use the classifier to eliminate the bad ones. Currently our matching criteria extracts as low as 79% of all good matches, which bounds the recall of the system. We are experimenting with different string distance metrics proposed by Cohen et al. [7] to try and raise the number of good matches returned.

## Figures and Tables

**Table 1:** Example of partial entries from the fly synonym list.

Normalized Form	Possible Synonyms
FBgn0003943	CG11624 Ub, Ubi p, Ubi63E, polyubiquitin
FBgn0003944	CG10388 Cbx, DmUbx, Hm, Ubx, abx, bithorax
FBgn0003945	Udg, Uracil DNA glycosylase
FBgn0004837	Suppressor of Hairless, br7, C: Group C, RBP JKappa, lethal 7 in the black-reduced region

**Table 2:** Performance of pattern matching system on development data. Precision and recall numbers for pattern matching system using: **A) **Simple direct matching of synonyms to text. **B) **Direct matching of synonyms to text only considering informative synonyms. **C) **Same as B, except restrict that a synonym must be in a documents candidate list for match to be valid. **D) **Same as C, except matches are run with all tokens stemmed. Numbers are reported for both fly and mouse.

**Fly**	**Precision**	**Recall**	**F-measure**
A. basic matching	0.033	0.861	0.063
B. informative syns	0.458	0.727	0.562
C. candidate list	0.709	0.667	0.687
D. stemming	0.713	0.690	0.701

**mouse**	**Precision**	**Recall**	**F-measure**

A. basic matching	0.151	0.583	0.240
B. informative syns	0.478	0.548	0.511
C. candidate list	0.739	0.505	0.600
D. stemming	0.716	0.656	0.685

**Table 3:** Pattern matching performance on evaluation data.

**Organism**	**Precision**	**Recall**	**F-measure**
fly	0.638	0.695	0.665
mouse	0.830	0.673	0.743
yeast	0.950	0.894	0.921

**Table 4:** Maximum entropy classification performance on evaluation data.

**Organism**	**Precision**	**Recall**	**F-measure**
fly	0.704	0.783	0.742
mouse	0.787	0.732	0.758
yeast	0.956	0.881	0.917
